# Viable Cryopreservation Strategy for Extending the Timeframe of Circulating Tumor Cell Detection in Breast Cancer Clinical Trials

**DOI:** 10.3390/biom15050723

**Published:** 2025-05-15

**Authors:** Cristina Sánchez-Quesada, Estefanía Toledo, José Juan Jiménez-Moleón, José Juan Gaforio

**Affiliations:** 1 Department of Health Sciences, Faculty of Health Sciences, University of Jaén, 23071 Jaén, Spain; csquesad@ujaen.es; 2 University Institute of Research on Olive and Olive Oils (INUO), University of Jaen, Campus las Lagunillas s/n, 23071 Jaén, Spain; 3 Agrifood Campus of International Excellence (ceiA3), 14071 Córdoba, Spain; 4 Department of Preventive Medicine and Public Health, University of Navarra, C/Irunlarrea, 1, 31008 Pamplona, Spain; etoledo@unav.es; 5 IdiSNA, Navarra Institute for Health Research, 31008 Pamplona, Spain; 6 CIBERobn Physiopathology of Obesity and Nutrition, Institute of Health Carlos III (ISCIII), 28029 Madrid, Spain; 7 Department of Preventive Medicine and Public Health, University of Granada, 18071 Granada, Spain; jjmoleon@ugr.es; 8 Instituto de Investigación Biosanitaria ibs.GRANADA, 18012 Granada, Spain; 9 Consortium for Biomedical Research in Epidemiology and Public Health (CIBERESP), Institute of Health Carlos III (ISCIII), 28029 Madrid, Spain

**Keywords:** tumor cell, immunodetection, fluid biopsy, liquid biopsy

## Abstract

Circulating tumor cells (CTCs) hold recognized prognostic value in various cancers, including breast cancer, where their presence correlates with survival outcomes. However, the typical 24 h window for blood processing and CTC isolation poses a logistical challenge, particularly for multicenter studies. This study aimed to evaluate cryopreservation at different stages of CTC isolation and immunocytological detection to extend the blood sample processing period. Using spiked peripheral blood samples with MDA-MB-231, SKBR3, and MCF7 breast cancer cell lines, four distinct cryopreservation points were assessed: following Ficoll gradient separation, immunomagnetic separation, cytocentrifugation, and cytokeratin labeling. Our findings demonstrated that cryopreservation of the mononuclear and granulocytic cell fraction after double-density Ficoll gradient separation was the only viable method for subsequent CTC detection. This approach allowed for consistent recovery of CK+ CTCs, with an average recovery rate of over 81% after one year of cryopreservation. In contrast, cryopreservation at later stages resulted in undetectable CTCs or only cellular debris. In conclusion, cryopreservation following density gradient centrifugation is a feasible strategy for delaying CTC isolation and immunocytological analysis in breast cancer research, facilitating its application in multicenter clinical trials.

## 1. Introduction

In 1869, an observant researcher named Ashworth made a significant discovery [1]. While studying the blood of a patient with metastatic cancer, Ashworth noticed the presence of “some cells” that bore a striking resemblance to the tumor cells found in the patient’s primary tumor [1]. This was the first documented observation of what we now know as circulating tumor cells (CTCs), cancer cells that have detached from the original tumor and entered the bloodstream [2]. Ashworth’s initial finding suggested that these circulating cells could be the “substrate of metastasis”, playing a pivotal role in the dissemination of cancer to distant sites. However, due to the inherent technical challenges in isolating these rare cells from the overwhelming number of blood cells, it took more than a century for researchers to fully appreciate the critical role of CTCs in cancer metastasis [1]. Despite this early observation, it was not until the development of emerging technologies in the past two decades that the study of CTCs significantly advanced, allowing for a deeper understanding of their biology and clinical applications.

CTCs are considered the “substrate of metastasis”, and their study provides invaluable insights into the metastatic cascade [1]. Research involving the injection of patient-derived CTCs into mice has elucidated intrinsic properties of these cells, such as the role of SEMA4D (semaphorin 4D) in breaching the blood–brain barrier [2]. Epithelial–mesenchymal transition (EMT) in CTCs, often indicated by TWIST1 upregulation and decreased E-Cadherin expression, is associated with enhanced metastatic capacity [1,2]. The presence of specific markers like uPAR and integrin β1 on EpCAM-negative CTCs has also been linked to brain metastasis. Furthermore, studies have shown that only certain CTCs exhibit tropism for specific organs like the brain, bone, or liver [2].

Clinical trials are investigating the utility of CTCs in guiding treatment decisions. The STIC CTC trial, for instance, showed that a CTC-based choice of first-line therapy in HER2-negative, hormone-receptor-positive metastatic breast cancer was non-inferior to the clinician’s choice [3,4]. Identifying molecular characteristics of CTCs, such as HER2 expression, could potentially personalize treatment [5].

Circulating tumor cells (CTCs) are recognized as possessing prognostic value in a variety of cancers [6,7,8,9]. The study of CTC appearance and dissemination has been proposed as a valuable tool for guiding cancer therapy and monitoring patients with cancer [10]. In 2007, the American Society of Clinical Oncology initially highlighted the clinical utility of CTCs [11]. Specifically in breast cancer, the presence of CTCs has been shown to correlate with progression-free survival and overall survival [6]. Currently, CTCs are acknowledged as a significant prognostic factor for relapse or mortality in several cancers [5]. Cristofanilli’s study demonstrated that both CTC levels and their fluctuations over time can identify high-risk patients with metastatic breast cancer who could potentially benefit from early therapeutic modifications [12].

The isolation and detection of CTCs are performed using liquid biopsies, which are minimally invasive blood tests designed to detect CTCs. Current CTC enrichment and isolation techniques include immunoaffinity-based methods, size-based methods, density-based methods, and combinations thereof [13]. The clinical application of a high-throughput microfluidic device for efficient and semi-automated enrichment of large numbers of CTCs from leukopaks of metastatic cancer patients has been evaluated. Overall, microfluidic platforms offer the advantage of easy scalability and automation, with the potential to create point-of-care devices for clinical applications in cancer monitoring [14].

Detecting these rare CTCs among the numerous normal blood cells is challenging; however, emerging technologies have significantly advanced the field [1,4]. These include EpCAM-based methods like CellSearch [4], as well as marker-independent techniques based on physical properties like size and deformability and nanotechnology-based approaches using microfluidic chips and nanoparticles [1]. Single-cell sequencing of CTCs allows for detailed analysis of their genome and transcriptome, revealing heterogeneity and potential drug targets [1].

Nevertheless, three main methods are currently employed for CTC detection: immunocytology (as immunocytochemistry or flow cytometry), biomolecular techniques (qRT-PCR, PCR), and functional assays (EPISPOT, EPIDROP) [15]. However, CTCs’ quantification combined with genetic analyses (e.g., deletion, polysomies) could provide a more specific description of the progress and prognosis of cancer patients [16]. Immunocytology also offers the advantage of allowing for the characterization of isolated CTCs for further analyses [17].

CTCs are expected to be a crucial component of “Precision medicine” [1]. Their phenotypic, genotypic, and functional characterization can provide opportunities to study drug susceptibility related to metastasis and unveil potential drug targets [1]. While challenges like isolating viable CTCs for culture persist, ongoing research and technological advancements continue to underscore the vital role of CTCs in understanding and managing cancer [5].

Despite recent advances in immunocytological detection methods, a key challenge remains the limited optimal timeframe for blood processing and CTC isolation, typically within 24 h of blood draw [18]. However, this short timeframe for sample processing may limit the application of CTC detection in translational clinical trials or routine clinical care, as a short time to transfer blood samples to specialized centers of analysis would be needed. Therefore, the development of new techniques to delay CTC analyses would be advantageous by enabling CTC detection in settings where processing within 24 h is not feasible, such as multicenter studies. Delaying CTC isolation from peripheral blood (PB) could be a valuable strategy in multicenter clinical trials where this process is centralized in a single laboratory to minimize bias. Identifying a method to allow for PB collection and subsequent CTC isolation at another location could facilitate CTC analysis for studying individual outcomes of breast cancer patients, particularly given that CTC level fluctuations can identify potential high-risk patients with metastatic breast cancer [6,12]. Until the present study, no validated method existed.

One alternative for delaying CTC analysis is the use of fixative protocols for PB, which could provide several days for processing [19]. However, common fixatives may interfere with downstream molecular analyses. Cryopreservation offers another alternative, allowing for delayed CTC detection and subsequent analysis. One advantage of cryopreservation is the minimal local processing, followed by frozen transport and feasible cryostorage. Furthermore, cryopreservation allows for further phenotypic and genetic analyses of these cells, which could include staining, quantification, or sequencing, as shown for fresh blood samples [20]. Cryopreservation has been used in several cancers [21,22], but, to our knowledge, no previous work has described cryopreservation for delayed CTC analyses in breast cancer cells from an experimental model in vitro.

The aim of this study was to assess if cryopreservation could be used for delaying CTC isolation and detection through immunocytology methods and to find the best protocol step to do it in order to enhance the time for processing the liquid biopsy sample.

## 2. Materials and Methods

### 2.1. Chemicals

Minimum Essential Medium (MEM) with Eagle’s salts was purchased from Gibco Life Technologies Ltd. (Paisley, UK), while RPMI 1640 and Fetal Bovine Serum (FBS) were obtained from PAA Laboratories GmbH (Pasching, Austria). EDTA tubes (BD-Vacutainer) were purchased from Becton Dickinson (Heidelgerg, Germany). HEPES buffer, sodium pyruvate, non-essential amino acids (NEAA), Histopaque 1119, Histopaque 1077, Fast Red TR/Naphthol AS-MX substrate solution, and poly-L-lysine-coated glass slides were obtained from Sigma-Aldrich (St. Louis, MO, USA). The Carcinoma Cell Enrichment and Detection Kit and MACS Columns were purchased from Miltenyi Biotec (Bergisch Gladbach, Germany). Kaiser’s glycerol gelatin was acquired from Merck (Darmstadt, Germany). Dimethyl sulfoxide (DMSO) was purchased from Panreac Applichem (Darmstadt, Germany).

### 2.2. Cell Line and Blood Control Samples

MDA-MB-231, SKBR3, and MCF7 cell lines were obtained from the American Type Culture Collection (ATCC; Rockville, MD, USA). MDA-MB-231 and MCF7 cells were cultured in MEM supplemented with 10% heat-inactivated FBS and 1% of each of HEPES buffer, sodium pyruvate, and NEAA. SKBR3 cells were cultured in RPMI 1640 supplemented with 10% heat-inactivated FBS. Cells were cultured as monolayers at 37 °C in a 5% CO_2_ atmosphere. Cells in the exponential growth phase were used for all experiments. These cancer cells were used as a positive control.

Peripheral venous blood samples (10 mL) were collected from two healthy donors following an approved ethical protocol and with signed informed consent. Samples were collected in EDTA tubes and processed within 6 h of collection. The initial milliliter was discarded to minimize epithelial cell contamination.

For positive controls, 250 tumor cells were spiked into 10 mL of PB from healthy donors. Previously, we validated the absence of CTCs in PB from these healthy donors as negative controls. Positive and negative controls were performed separately to prevent cross-contamination. Subsequently, the positive and negative blood controls in EDTA tubes were kept at 4 °C prior to further analysis.

### 2.3. Isolation and Immunodetection of CK+/CTCs Through Magnetic Separation Without Cryopreservation

CTC detection and isolation using immunomagnetic separation were performed according to a methodology previously described by our group [23]. Briefly, a double-density Ficoll gradient was prepared for each 10 mL PB sample, consisting of 5 mL of Histopaque 1119 and 5 mL of Histopaque 1077. Following centrifugation (700× *g*, 30 min, 20−25 °C), the mononuclear and granulocyte fraction was isolated and then centrifuged in 20 mL of PBS. The supernatant was discarded, and the cell pellets were permeabilized with 5 mL of Cell-Perm solution for 5 min, followed by fixation with 5 mL of Cell Fix solution for 30 min, according to the manufacturer’s instructions (Carcinoma Cell Enrichment and Detection Kit, Miltenyi Biotec). Following centrifugation of the cell suspension at 300× *g* for 10 min, the cells were resuspended in 600 µL of 1× MACS Cell Stain Solution. Subsequently, 200 µL of FcR Blocking reagent was added and mixed thoroughly with 200 µL of MACS Cytokeratin Microbeads. Samples were incubated for 45 min at 20–25 °C. To detect and quantify cytokeratin-expressing tumor cells through immunocytology, 100 µL of Anti-Cytokeratin-FITC was added, and the samples were incubated for an additional 15 min in the dark at 20–25 °C. 4 mL of 1× MACS CellStain SolutionA volume of 4 mL of 1× MACS CellStain Solution *g*was added, and the cell suspension was centrifuged at 300× *g* for 10 min. The supernatant was removed completely, and the cell pellet was resuspended in 500 µL of 1× MACS CellStain Solution. Then, 10 μL of Anti-FITC Alkaline Phosphatase was added and incubated for 15 min in the dark at 20−25 °C. Afterward, 490 µL of 1× MACS CellStain Solution was added, and the cells were submitted to immunomagnetic separation with a MS+/RS+ positive selection column (Miltenyi Biotec). The magnetically enriched cell fraction was cytocentrifuged onto a slide using a centrifuge (Hettich; Tuttlingen, Germany). Slides were air-dried for 2–24 h at 20–25 °C. Finally, the slides were washed once for one minute in PBS. Then, 50 µL of freshly prepared Fast Red TR/Naphthol AS-MX Substrate Solution was added to the cell spots on the slides, and they were incubated for 15 min in a humidity chamber at 20–25 °C. Slides were washed for one minute in double-distilled water and thereafter air-dried. Slides were mounted with Kaiser’s glycerol gelatin [23].

### 2.4. Cryopreservation of CTCs

**Figure 1:**
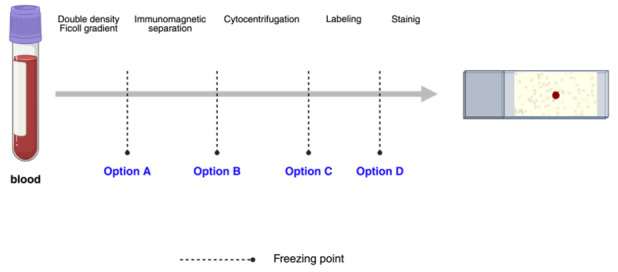
Isolation and enrichment of circulating tumor cells (CTCs) through peripheral blood scheme.

Cell cryopreservation was evaluated at four distinct stages of the procedure detailed in Section 2.3: (A) following double-density Ficoll gradient separation, (B) following immunomagnetic separation, (C) following cytocentrifugation, and (D) following labeling (Figure 1). For each option, three blood samples containing MDA-MB-231 tumor cells, three samples with SKBR3, and three samples with MCF7 were analyzed. A total of 36 positive controls and 12 negative controls were assayed across the four options (A, B, C, and D).

All samples were cryopreserved in two steps. Firstly , samples were frozen at −20 °C for 10 min, and, secondly , they were stored at −80 °C for a minimum of 24 h before proceeding with the protocol for the isolation and immunodetection of CK+/CTCs.

**Figure 2:**
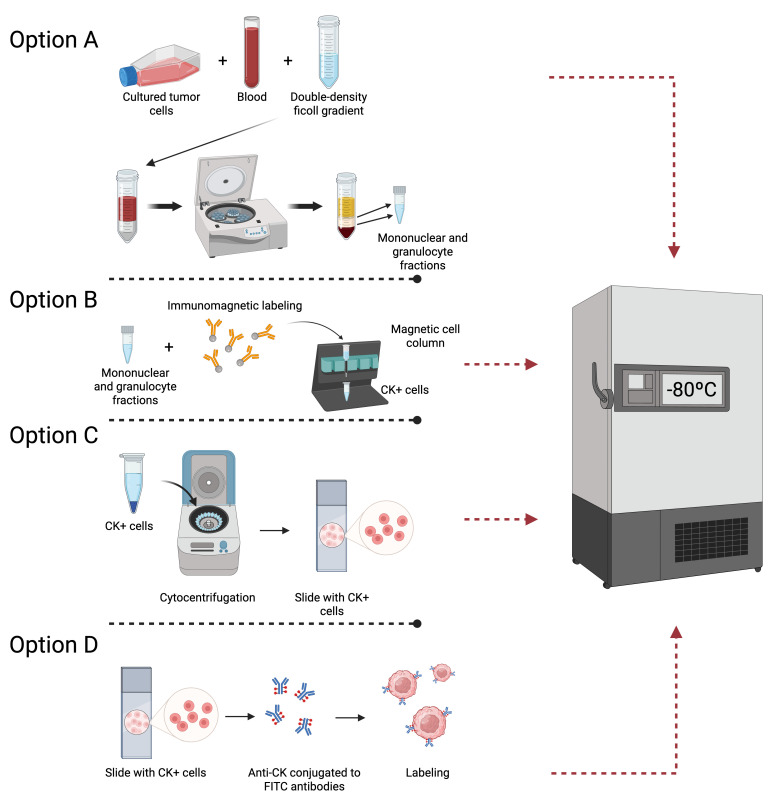
Possible cryopreservation options throughout the isolation and enrichment of CTC. (**A**) Cryopreservation after double-density Ficoll gradient, (**B**) cryopreservation after immunomagnetic separation, (**C**) cryopreservation after cytocentrifugation, and (**D**) cryopreservation after cytokeratin labeling.

In option A, 10 mL of peripheral venous blood samples was collected. Then, a double-density Ficoll gradient was prepared for each PB sample, and the mononuclear and granulocytic cell fraction were isolated and centrifuged in PBS (Figure 2A). The supernatant was discarded, and the cells were transferred to 1 mL sterile cryopreservation tubes containing a mixture of FBS and 10% DMSO.

In option B, samples were permeabilized with Cell-Perm solution and fixed with Cell Fix solution. Subsequently, FcR Blocking reagent was used, and the samples were mixed with MACS Cytokeratin Microbeads. Cells were submitted to immunomagnetic separation (Figure 2B), and CK+ cells were eluted and centrifuged in MACS Cell Stain Solution. Afterward, cells were placed into sterile cryopreservation tubes with a mixture of FBS and DMSO at a concentration of 10%.

In option C, after the magnetically enriched CK+ cell fractions were cytocentrifuged onto poly-L-lysine-coated glass slides and air-dried overnight at 20–25 °C, the slides were stored at −80 °C without fixation (Figure 2C).

In option D, slides were labeled with Anti-CK- FITC and Anti-FITC Alkaline Phosphatase. Subsequently, the samples were stored at −80 °C until further procedures (Figure 2D).

### 2.5. Implementation of Cryopreservation Duration

Following the determination of the optimal stage for cell cryopreservation that enables the preservation of CTCs, we evaluated various freezing durations (1 week, 1 month, 4 months, 6 months, and 1 year) to ascertain the maximum storage time for frozen cells without compromising the subsequent isolation and quantification of CTCs through immunocytology. Consistent with prior experiments, we used samples with MDA-MB-231, MCF7, or SKBR3 tumor cells. All assays were performed in triplicate. A total of 45 positive controls were employed (9 samples for each freezing duration).

Finally, we tested the recovery and membrane integrity (color staining) after 1 year for MDA-MB-231, MCF7, and SKBR3 positive controls. CK+/CTC recovery was counted separately by two expert researchers in a blinded manner.

### 2.6. Statistics

The number of CK+/CTCs is presented as the mean of two counts by two independent researchers ± standard error of the mean (SEM). General variance analysis (ANOVA) and Student’s *t*-test were conducted. A *p* value < 0.05 was considered to be statistically significant. These statistical analyses were performed using Statgraphics Centurion XVIII statistical software (Statpoint Technologies, Inc., Warranton, VA, USA). Any statistically significant difference was found.

## 3. Results

### 3.1. Analysis of CTCs Through Immunocytology After Cryopreservation

Samples were collected within a maximum timeframe of 6 h. The time between collection and processing is essential to minimize cell death. Consequently, blood was processed within this strict 6 h window, which did not appear to affect cell mortality post-collection [6].

We analyzed cells from four distinct cryopreservation stages within the procedure—(A) following double-density Ficoll gradient separation, (B) following immunomagnetic separation, (C) following cytocentrifugation, and (D) prior to staining (Figure 2)—to determine morphological variation, whole-cell membrane integrity, and complete labeling and staining. We observed that only option A met all of the defined objectives. Recovery and membrane integrity were nearly perfect compared with the other alternatives. In options B, C, and D, we found significant debris, a noticeable absence of cells, very low recovery rates, or a failure of cells to stain.

In options B, C, and D, we did not detect CK+/CTCs in any sample of MDA-MB-231, MCF7, or SKBR3; we only detected red-stained cell debris. This lack of CTC detection may be due to difficulties in maintaining cell membrane integrity and, potentially, the cytoskeleton, as well. This is likely because, to label the CTCs, the cell membrane must be permeabilized to allow for the internalization of magnetic microbeads and labeling antibodies. It is possible that freezing after permeabilization, which is necessary to create these pores, makes the membrane unstable and thus causes it to disintegrate along with the cytoskeleton, resulting in the observed debris.

### 3.2. Analysis of Implementation of Cryopreserved Time

**Figure 3:**
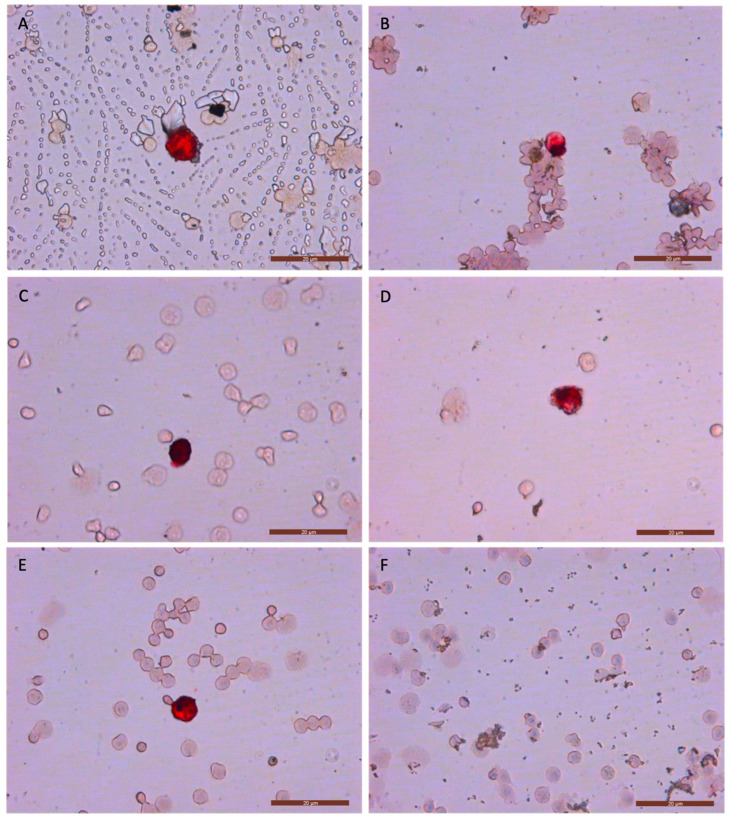
Representative images of circulating tumor cell (CTC) immunocytology of CK+/CTCs detected (red) and hematopoietic cells in positive controls after 24 h (**A**), 1 week (**B**), 1 month (**C**), 4 months (**D**), and 6 months (**E**) of freezing, and a negative control after 6 months of freezing (**F**).

To confirm that CTCs could be detected through immunocytology, we analyzed cells obtained following double-density gradient separation with Ficoll (option A) after different freezing time points: 24 h, 1 week, 1 month, 4 months, 6 months, and 1 year. We found that all CK+/CTCs detected in samples from each frozen time point were well-defined and stained red (Figure 3 and Supplementary Materials). However, cell aggregates appeared in panels B and E2. These aggregates may have been caused by suboptimal handling during the implementation of the technique, including, specifically, difficulties in separating the cells after centrifugation. Furthermore, the air bubbles observed in panel A were probably due to poor mounting of the sample slide.

### 3.3. Analysis of CK+/CTC Recovery in All Frozen Time Points

We performed an analysis of recovery in positive controls at each tested frozen time point. Analysis after one year of cryopreservation using option A of the protocol confirmed that the cells maintained their morphological characteristics. The total recovery of CK+/CTCs was 82.6% (mean recovery: 206.5 ± 9.5) for MDA-MB-231 positive controls, 79.4% (198.5 ± 4.4) for SKBR3, and 81.5% (203.7 ± 6.3) for MCF7.

The recovery of CK+/CTCs was enhanced compared with previously published data by our group [6]. In 2003, Gaforio et al. reported a recovery range of 60–80% for breast cancer cells spiked into positive controls, while this procedure achieved a maximum of 82.6%. Considering this, future modifications to the protocol to enhance CTC recovery in PB samples from breast cancer patients will be of interest.

## 4. Discussion

The earliest indications of CTCs date back to 1869, when the Australian pathologist Thomas Ashworth observed cells resembling those of the primary tumor in the bloodstream of patients with metastatic disease [1]. Although not specific to breast cancer, this marked the first documented observation of what we now understand as CTCs. However, limited technology hindered further investigation, and the significance of these findings was not immediately realized.

For much of the 20th century, research into CTCs was significantly hampered by their scarcity in peripheral blood, surrounded by an overwhelming number of normal blood cells. Detecting these elusive cells necessitated laborious methods and lacked the requisite sensitivity for meaningful clinical applications. A pivotal moment in the history of CTC research in breast cancer was the pioneering description by Gaforio et al. [6], who described for the first time that their presence in blood correlated with progression-free survival and overall survival. Based on this, the 2007 update of the American Society of Clinical Oncology guidelines for the use of tumor markers in breast cancer proposes CTCs as a new marker for breast cancer [11]. Subsequently, novel technologies were developed for the detection of CTCs, such as the CellSearch^®^ system, the first FDA-approved method for CTC isolation. This system, utilizing immunomagnetic capture based on the EpCAM (epithelial cell adhesion molecule) antigen and identification via fluorescently labeled antibodies against cytokeratins (CK), enabled large-scale studies and the establishment of the clinical validity of CTCs [4].

The seminal 2004 study by Cristofanilli et al. demonstrated that a baseline CTC count (≥5 CTCs/7.5 mL) was an independent prognostic factor for overall survival and progression-free survival in patients with metastatic breast cancer [12]. Subsequent studies independently corroborated this observation. Measuring CTC dynamics over time also proved more informative than a single measurement. A threshold of ≥5 CTCs/7.5 mL of blood was established to define a high count in the metastatic context. The AJCC Cancer Staging Manual even identifies an aM0(i+) category for breast cancer, defined by detected tumor cells in circulation without clinical or radiographic evidence of distant metastases [4].

Subsequent to the validation of CTCs in metastatic breast cancer, research expanded to early breast cancer. Studies in neoadjuvant settings showed that the presence of CTCs before neoadjuvant chemotherapy was associated with poorer disease-free survival and overall survival. A meta-analysis further solidified CTCs as a strong quantitative and independent prognostic factor in this context [4]. In the adjuvant setting, trials like TREAT-CTC (EORTC 90091-10093) investigated the role of trastuzumab in HER2 non-amplified early breast cancer with circulating tumor cells, but it was stopped for futility, highlighting the complexity and the need for molecular characterization of CTCs [5]. Lower CTC thresholds (≥1 CTC/7.5 mL or even ≥1 CTC/15 mL) were considered relevant in the early breast cancer setting.

As technology advanced, research shifted towards a more in-depth understanding of CTCs’ biology in breast cancer and their role in metastasis. Markers of epithelial-to-mesenchymal transition (EMT), such as TWIST1 and the loss of E-Cadherin, were investigated, associated with increased metastatic potential [1]. The expression of other markers on CTCs was also explored to subclassify these cells and predict their tropism to different organs. For example, the role of SEMA4D (semaphorin 4D) in extravasation and blood–brain barrier transmigration was revealed [2].

The crucial next step was to ascertain whether the information provided by CTCs could guide treatment decisions in breast cancer. Several clinical trials were conducted with this goal. The SWOG S0500 trial (NCT00382018) attempted to improve outcomes through early switching of chemotherapy in metastatic breast cancer patients with persistently elevated CTCs after the first cycle, but it did not demonstrate a significant survival benefit [3]. However, the STIC CTC trial (NCT01710605) suggested that choosing first-line therapy (chemotherapy vs. endocrine therapy) based on CTC counts in HER2-negative, hormone-receptor-positive metastatic breast cancer patients was non-inferior to the physician’s choice and could offer benefits in certain subgroups. Trials like CirCe01 (NCT01401103) and CirCe T-DM1 also explored CTC-driven treatment strategies in later lines of MBC therapy, demonstrating limited success in improving overall survival [4].

In recent years, numerous novel technologies for CTC detection and characterization in breast cancer have emerged, including microfluidic-based and nanotechnology-based approaches [1]. These technologies allow for more detailed analysis of CTCs at the single-cell level, including DNA and RNA sequencing and proteomic analysis [4]. The future of CTCs in breast cancer likely lies in multi-parametric approaches integrating CTC analysis with other components of liquid biopsy, such as circulating tumor DNA (ctDNA) and exosomes [24]. These combined approaches could provide a more comprehensive understanding of the disease and its evolution, paving the way for personalized medicine and the development of targeted therapies.

This study has validated a procedure that enables the isolation and quantification of CTCs for up to 12 months after blood collection through the implementation of cryopreservation following the initial peripheral blood mononuclear cell density gradient separation. The analysis of CTCs has been suggested to be a valuable predictive tool in patients with solid tumors [25]. CTC analysis, performed on a small volume of blood obtained via a minimally invasive procedure, can provide valuable information for both the detection and characterization of minimal residual disease [13].

The presence of CTCs is currently determined using molecular biology techniques (e.g., RT-PCR), immunocytological methods (e.g., immunocytochemistry, flow cytometry), or functional assays (e.g., EPISPOT assay/EPIDROP) [15]. While molecular techniques, such as RT-PCR, are commonly employed for CTC detection [15,26], immunocytology remains the gold standard [17], primarily because it offers the significant advantage of enabling the genetic and morphological characterization of individual isolated CTCs. A key limitation of CTC immunocytology is the requirement for sample processing within 24 h of blood collection [18], which poses a challenge for studies and clinical practice in settings without immediate access to a specialized CTC analysis laboratory. Therefore, strategies to delay CTC isolation and analysis are highly desirable.

This study highlights the feasibility of performing immunocytological analysis, isolation, and quantification of CTCs in both multicenter clinical trials and in medical centers without an on-site specialized clinical laboratory.

As demonstrated by Cristofanilli et al. [12], CTCs represent a significant prognostic factor for relapse or mortality in various cancers. Importantly, not only CTC levels but also their fluctuations over time can identify high-risk patients with metastatic breast cancer who may benefit from early therapeutic adjustments. A technique enabling the observation of CTC number fluctuations during treatment could contribute to improved overall survival in breast cancer patients by guiding timely therapeutic adjustments. Thus, confirming the feasibility of immunocytological labeling of CTCs after cryopreservation represents a valuable asset, particularly in multicenter clinical studies, and a significant advancement in liquid biopsy immunoassays.

Following the isolation and quantification of CTCs cryopreserved at various stages of the isolation process from peripheral blood mononuclear cells, our findings indicate that cryopreservation of the cell fraction obtained after double-density Ficoll gradient separation (option A, Figure 2A) is the most efficacious approach. At all other tested cryopreservation points, we found CTCs to be undetectable, or we observed only cellular debris. The recovery rate of CTCs after cryopreservation in option A was 82.6%, demonstrating the suitability of this method for CTC enumeration. This is consistent with our previously published findings [6], which reported CTC recovery rates ranging from 60% to 80%. Interestingly, the number of CTCs was associated with tumor size (particularly cT4d inflammatory tumors) but not with pathological complete response. Most importantly, a higher CTC count had a detrimental effect on distant disease-free survival (*p* < 0.001), locoregional relapse-free interval (*p* < 0.001), and overall survival (*p* < 0.001). Indeed, the detection of CTCs before neoadjuvant chemotherapy independently predicted prognosis in multivariable analysis (notably independent of nodal status). Furthermore, CTC count as a continuous variable also held prognostic value for all endpoints, acting as a quantitative marker where each additional CTC detected correlated with a worse prognosis [4]. Hence, CTC count is of primary importance for studying disease-free survival and overall survival in breast cancer patients, and this novel procedure enables its analysis in both multicenter clinical trials and individual clinical centers.

To evaluate sample viability after prolonged cryostorage, we assessed different freezing time points using option A.

At each freezing time point, we analyzed the quality of labeling (color intensity and uniformity), morphological changes, and the recovery percentage (Figure 3). We observed no potential morphological changes, and the color intensity and uniformity were consistent with images from our previous studies [6,27], where fresh samples were analyzed. The recovery percentages, color intensity, and uniformity achieved with this new method support the idea of cryopreservation as an effective strategy for delaying CTC analysis in breast cancer. Furthermore, the immunocytology images of CTCs showed no appreciable differences compared with those obtained from fresh samples and even those from cancer patients in previous studies [6,23,27]. Additionally, we found this cryopreservation procedure and technique to be reproducible with the MDA-MB-231, MCF7, and SKBR3 cell lines after one year of cryopreservation (Supplementary Materials).

Although we have used only breast cancer tumor cells, it is possible that the above results may be applicable to other tumor types, although further studies are needed to confirm this.

## 5. Conclusions

In conclusion, cryopreservation of the mononuclear and granulocytic cell fraction isolated following density gradient centrifugation presents a viable strategy for the subsequent isolation and immunocytological analysis of CTCs in patients with breast cancer. Therefore, the next step is to implement this procedure in women diagnosed with breast cancer within a multicenter clinical trial, such as the LifeBreast trial (ClinicalTrials.gov Identifier: NCT04174391), to further evaluate CTC recovery as an outcome measure in breast cancer.

## Data Availability

Data that support the findings of this study are available from the corresponding author, J.J.G., upon reasonable request.

## Supplementary Materials

The following supporting information can be downloaded at https://www.mdpi.com/article/10.3390/biom15050723/s1, Figure S1: CK+/CTCs detected (red) and hematopoietic cells (brown) in positive controls of MCF7 cells (A) and SKBR3 cells (B) after 1 year of cryopreservation.

## Abbreviations

The following abbreviations are used in this manuscript:

CK+	Cytokeratin-positive
CTCs	Circulating tumor cells
RT	Room temperature
DMSO	Dimethyl sulfoxide
FBS	Fetal Bovine Serum
MEM	Minimum essential medium
NEAA	Non-essential amino acids
PB	Peripheral blood
