# Supplementary material for: Viable Cryopreservation Strategy for Extending the Timeframe of Circulating Tumor Cell Detection in Breast Cancer Clinical Trials
Source: Biomolecules. 2025 May 15;15(5):723. doi: 10.3390/biom15050723 (PMC12109437; doi:10.3390/biom15050723)
Supplement: Supplementary file 1 [file biomolecules-15-00723-s001.zip › biomolecules-3578284-supplementary.pdf]

Supplementary Materials

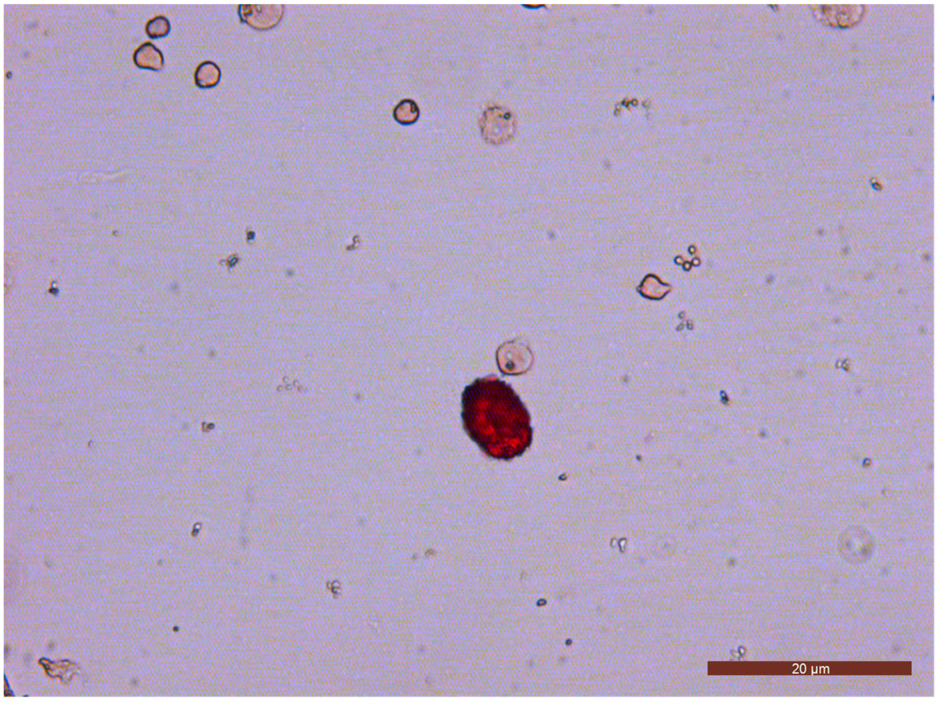

(**A**)

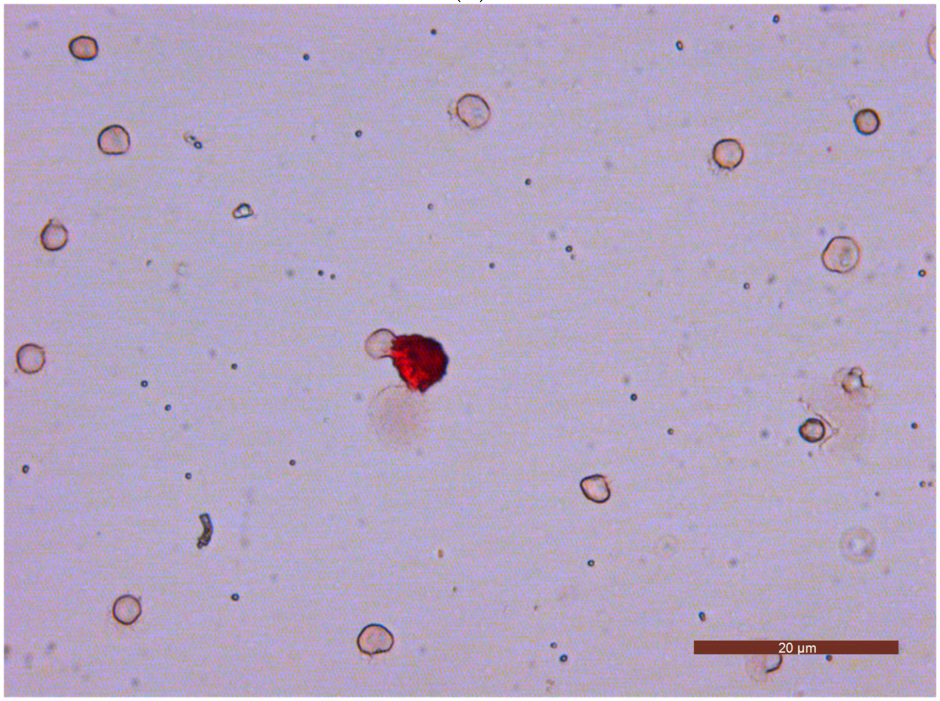

(**B**)

Figure S1. CK+/CTCs detected (red) and hematopoietic cells (brown) in positive controls of MCF7 cells (**A**) and SKBR3 cells (**B**) after 1 year of cryopreservation.
